# Influence of Heat Treatment and Reinforcements on Tensile Characteristics of Aluminium AA 5083/Silicon Carbide/Fly Ash Composites

**DOI:** 10.3390/ma14185261

**Published:** 2021-09-13

**Authors:** Santhosh Nagaraja, Ramesha Kodandappa, Khalid Ansari, Mohamed Saheer Kuruniyan, Asif Afzal, Abdul Razak Kaladgi, Navid Aslfattahi, C. Ahamed Saleel, Ashwin C. Gowda, Praveena Bindiganavile Anand

**Affiliations:** 1 Department of Mechanical Engineering, MVJ College of Engineering, Near ITPB, Whitefield, Bangalore 560067, India; 2 Department of Mechanical Engineering, School of Engineering and Technology, CHRIST (Deemed to be University), Bangalore 560074, India; ramesha.kra@gmail.com; 3 Department of Civil Engineering, Yeshwantrao Chavan College of Engineering, Nagpur 441110, India; khalidshamim86@rediffmail.com; 4 Department of Dental Technology, College of Applied Medical Science, King Khalid University, P.O. Box 394, Abha 61421, Saudi Arabia; mkurunian@kku.edu.sa; 5 Department of Mechanical Engineering, P. A. College of Engineering (Affiliated to Visvesvaraya Technological University, Belagavi), Mangaluru 574153, India; arkmech9@gmail.com; 6 Department of Mechanical Engineering, School of Technology, Glocal University, Delhi-Yamunotri Marg, SH-57, Mirzapur Pole, Saharanpur 247121, India; 7 Department of Mechanical Engineering, Faculty of Engineering, University of Malaya, Kuala Lumpur 50603, Malaysia; navid.fth87@yahoo.com; 8 Department of Mechanical Engineering, College of Engineering, King Khalid University, P.O. Box 394, Abha 61421, Saudi Arabia; ahamedsaleel@gmail.com; 9 Department Studies of Mechanical Engineering, Visvesvaraya Technological University, Muddenahalli, Chikkaballapura 562103, India; acgmech@gmal.com; 10 Department of Mechanical Engineering, Nitte Meenakshi Institute of Technology, Bangalore 560064, India; praveen.ba@nmit.ac.in

**Keywords:** heat treatment, AA 5083 alloy, silicon carbide, fly ash, composites, tensile, characteristics, sandblasting, composite, denture base

## Abstract

The effect of reinforcements and thermal exposure on the tensile properties of aluminium AA 5083–silicon carbide (SiC)–fly ash composites were studied in the present work. The specimens were fabricated with varying wt.% of fly ash and silicon carbide and subjected to T6 thermal cycle conditions to enhance the properties through “precipitation hardening”. The analyses of the microstructure and the elemental distribution were carried out using scanning electron microscopic (SEM) images and energy dispersive spectroscopy (EDS). The composite specimens thus subjected to thermal treatment exhibit uniform distribution of the reinforcements, and the energy dispersive spectrum exhibit the presence of Al, Si, Mg, O elements, along with the traces of few other elements. The effects of reinforcements and heat treatment on the tensile properties were investigated through a set of scientifically designed experimental trials. From the investigations, it is observed that the tensile and yield strength increases up to 160 °C, beyond which there is a slight reduction in the tensile and yield strength with an increase in temperature (i.e., 200 °C). Additionally, the % elongation of the composites decreases substantially with the inclusion of the reinforcements and thermal exposure, leading to an increase in stiffness and elastic modulus of the specimens. The improvement in the strength and elastic modulus of the composites is attributed to a number of factors, i.e., the diffusion mechanism, composition of the reinforcements, heat treatment temperatures, and grain refinement. Further, the optimisation studies and ANN modelling validated the experimental outcomes and provided the training models for the test data with the correlation coefficients for interpolating the results for different sets of parameters, thereby facilitating the fabrication of hybrid composite components for various automotive and aerospace applications.

## 1. Introduction

The investigation on the necessity for materials for explicit applications to support the interaction capacities has assisted the examination of the most recent advancements for the evolution of composites. Aluminium composites are used profusely in aircraft owing to the higher strength-to-weight ratio, especially with regard to tensile and yield strength, along with the corrosion resistance characteristics [1,2]. These composites have the ability to deal with high temperatures, especially for high-speed flight conditions. Subsequently, they have extended the scope for the investigation of the composites for their utilisation in aircraft flying at high Mach numbers that are exposed to higher temperatures in the range of 200–250 °C. Further, the examination of thermal exposure of aluminium composites to greater temperatures is very vital for aerospace applications, i.e., wing structures, airframes, etc. [3,4,5]. The aluminium alloys have incredible attributes particularly concerning castability, corrosion resistance, and high strength characteristics [6,7,8]. The attributes of the aluminium castings synthesised en route stir casting have gained momentum especially for high-temperature applications [9,10]. Aluminium oxide particles are commonly used as a sandblasting media, particularly in dentistry, for multiple purposes, including divesting the casting investment materials and increasing effective surface area for enhancing the mechanical retention strengths of succeeding applied fired porcelain or luting cements. Aluminium is used as denture base material, and its ceramics are also used for making crowns [11,12]. Zirconia-fused alumina metals are used for making dentures, crowns, and bridges for artificial dentures. Mainly base metal alloys and noble metal alloys are used. These metals are polished and finished after casting. Zirconia-fused alumina metals are used for making bonded abrasives products and in the processes of grinding, sandblasting, and surface treatment of metal products and other materials [13,14].

The composites essentially have two constituents, i.e., the hypo-eutectic blends of Al alloys and the reinforcements. The morphology of the carbide particles is pivotal for deciding the tensile characteristics of the composites [15,16]. The composites are exposed to heat treatment as per the T6 temper conditions, which are known to eventually increase the tensile strength of the composites [17,18,19,20]. In connection with the effect of heat treatment on the characteristics of the composites, Reddy et al. [21] synthesised composites from AA 7075 as the matrix phase reinforced with the E-glass and fly ash reinforcements, en route stir casting and solution treatment, subsequently followed by water quenching; it was found that heat treatment enhances the tensile characteristics of the AA 7075 alloy composites due to Orowan strengthening and strong bonding. Garg et al. [22] fabricated aluminium alloy composites by varying the wt.% and the particle size and found that the increase in the fly ash size with heat treatment results in an increase in tensile strength due to grain refinement and grain involution in the matrix phase. Gangil et al. [23] studied the effect of heat treatment on the properties of the aluminium–fly ash–SiC hybrid composites and reported that the properties of the composites improve with the inoculation of the reinforcements in the composites. Chak et al. [24] reviewed the effect of rotational speed and temperature in stir casting and thermal exposure on the characteristics of Al-7Si-0.35Mg alloy matrix reinforced with varying wt.% of Al_2_O_3_ in the range of 2 to 8 and reported that the tensile characteristics improve due to particle diffusion and particle–matrix bonding. Bienia et al. [25] carried out a characteristic study on the aluminium–SiC composites and evaluated the characteristics of the composites; they reported that the properties of the composites improved with the increment in fly ash content, subsequently followed by the micro coring and segregation in the matrix. Suragimath et al. [26] fabricated LM-aluminium-alloy-based composites by reinforcing with SiC and fly ash and reported that the tensile characteristics increase with the increment in the fly ash composition, thereby ascertaining the influence of composition and thermal exposure on the composites.

Rohatgi et al. [27] conducted extensive research on the effect of reinforcements on the aluminium matrix composites and reported that the tensile characteristics decrease with the increment in the reinforcement particle size; however, the increase in heat treatment temperature by 20 °C increases the strength characteristics of the composites owing to Orowan strengthening brought about by strong bonding and grain growth inoculation. Baradeswarn et al. [28] carried out extensive work on the effect of reinforcements particle size on the composites and found that the compressive strength increases with an increase in wt.% of fly ash attributed to the coring and microsegregation of the particles in the matrix phase, leading to strengthening brought about by the epitaxial grain growth and interstitial bonding between the atoms. Rao et al. [29] found that the composites reinforced with graphite and alumina exhibit greater compression strength, ascribed to the effect of graphite which is a ceramic reinforcement, influencing the compression characteristics of the composites due to the effect of coring on the matrix composites. Rohatgi et al. [30] characterised the effect of cenospheres on AZ91D aluminium alloy and reported that the tensile properties increase with the inclusion of reinforcements in the matrix, thereby improving its metal workability characteristics for aerospace applications, thus providing an inference that the fly ash is having a limiting effect on the strength beyond 5 wt.%. Ozden et al. [31] examined the effect of heat treatment on the tensile characteristics. Two particle sizes of 160 µm and 520 µm of SiC and two extrusion ratios of 13:63:1 and 19:63:1 for AA 6063/SiC composites were selected. It was noticed that the SiC reinforced Al-6063 alloy composites show the highest tensile strength. The SEM images depict the uniform distribution of silicon carbide particles. From their findings, it can be concluded that various heat treatment temperatures on different aluminium-based alloys can be chosen for the enhancement of mechanical properties.

From the review of the research findings related to the scope of the present work, it is evident that the motivation for carrying out the research on the influence of thermal cycle on the mechanical properties of aluminium AA 5053–SiC–fly ash composites arises from the fact that the inclusion of the reinforcements and the treatment of the composites for the range of thermal cycle is found to enhance the tensile strength required for potential applications in automotive and aerospace components. Further, it is evident from the literature that there is sufficient scope for evaluating the effect of heat treatment on the properties of the aluminium composites, especially the effect of inoculation brought about by fly ash on the hybrid aluminium composites.

## 2. Materials and Methods

Aluminium AA 5083 alloys in billet form were procured from Perfect Metal Corporation, Bengaluru, Karnataka State, India, alongside SiC of an average size of 35 to 70 microns procured from Snam abrasives, Bengaluru, Karnataka State, India, and C-type, and fly ash procured from Karnataka Power Corporation, Raichur, Karnataka State, India, which were used as reinforcements. The selection of the matrix and reinforcement phases was carried out based on the property requirements and ground survey on the availability and requirements matrix; the composition and property of the matrix and reinforcements ascertained from the supplier specifications are given in Table 1, Table 2, Table 3 and Table 4, respectively.

**Figure 1:**
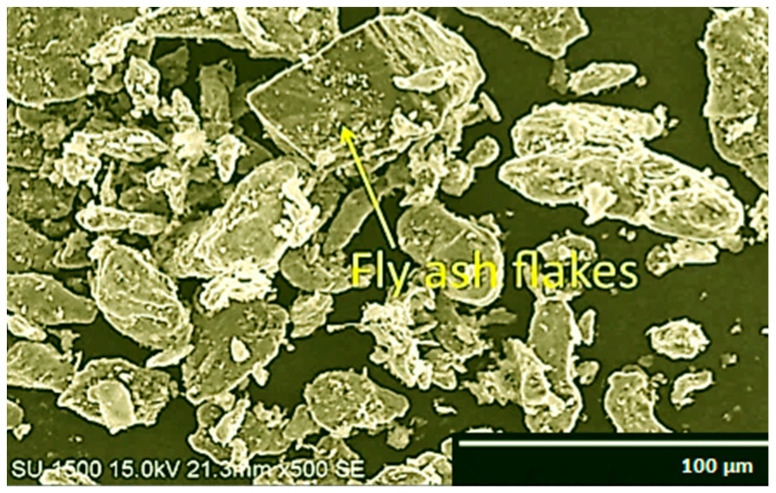
SEM of fly ash flakes at 500× magnification.

**Figure 2:**
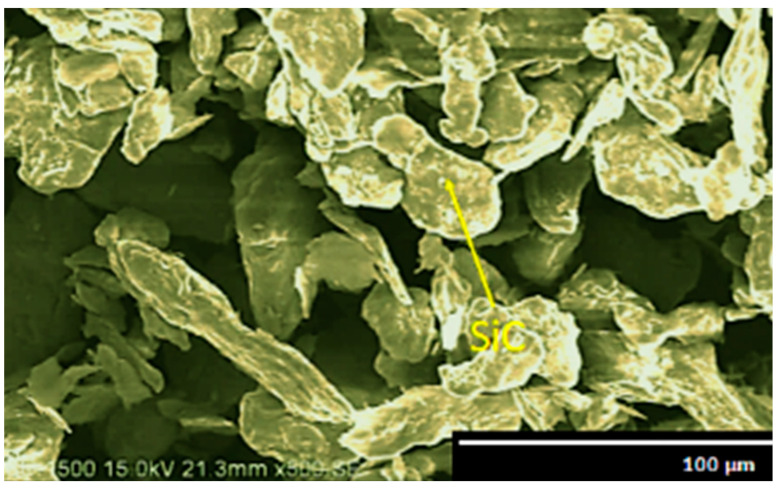
SEM of SiC at 500× magnification.

The micrographic images of fly ash flakes and SiC are observed using a Hitachi make SU-8000 SEM and their microstructural images are obtained at 500× magnification and 15 kV acceleration voltage. The size of fly ash flakes varies in the range of 45–125 µm (Figure 1), whereas the size of SiC varies in the range of 30–85 µm (Figure 2).

In SEM images, the particle size and shape of the fly ash and SiC are distinctly visible, and it is observed that the fly ash flakes and SiC are almost homogenous in size and have quasi-cubic and polyhedral sizes which are found to enhance the properties of the composite materials synthesised en route stir casting. The uniform dispersion of reinforcement particles can be attained by this shape of the particles and thus helps in strong bonding.

**Figure 3:**
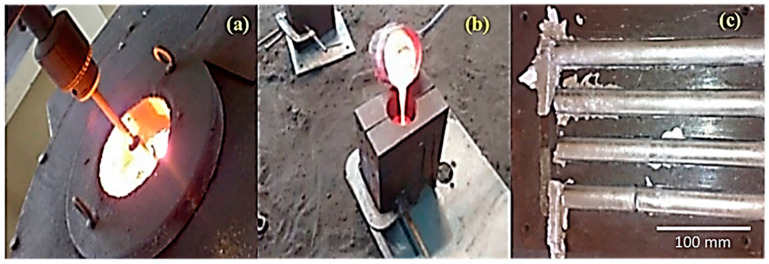
(**a**) Stir casting; (**b**) pouring of molten metal into die; (**c**) cast specimens.

The stir casting was accomplished to fabricate AA 5083-SiC-fly ash composites because of their capability to deliver high-performance composites. The weighed amount of cut pieces of aluminium AA 5083 were fed into the furnace, and the temperature was raised to 770 °C, following the works of Santhosh et al. [32,33]. The SiC and fly ash flakes were preheated to 350 °C for two and a half hours, added to the molten metal, and stirred using ceramic coated AISI 316L stirrer for a duration of 10 min at a speed of 600 rpm. Hexachloro–ethane (C_2_Cl_6_) tablets were plunged into the molten metal to remove the entrapped air. Subsequently, the temperature of molten metal was sustained at 750 °C, followed by a second round of stirring ceaselessly for a duration of 10 min before pouring the melt to the preheated die. The stir cast composites with varying wt.% of SiC and fly ash were subjected to varying temperatures, i.e., 40 °C, 80 °C, 120 °C, 160 °C, and 200 °C. The photographic images of the stir casting process, pouring of molten metal into the die-set, and cast specimens are given in Figure 3.

Table 5 gives the specimen designation, composition of the matrix, and the reinforcements for different specimens, and the temperature of exposure for the composite specimens fabricated.

### 2.1. T6-Grade Heat Treatment

The ‘T6-grade’ heat treatment cycle comprised of two stages, i.e., the solution treatment carried out at 530 °C for a duration of 2 h, followed by water quenching and ageing at a temperature of 150 °C for a duration of 6 h and the thermal treatment at temperatures of 40 °C, 80 °C, 120 °C, 160 °C, and 200 °C in Heattek make Oven, with time and temperature controls available at University, Visvesvaraya College of Engineering, Bangalore University, Bengaluru, Karnataka State, India, at 0.5 MPa operating pressure and 10 h soak time in still air, with time and temperature controls as per the AMS-2771 specifications. This process of solutionising, ageing, and thermal exposure facilitate the dissolution of intermetallic phases.

### 2.2. Characterisation for Tensile Properties

**Figure 4:**
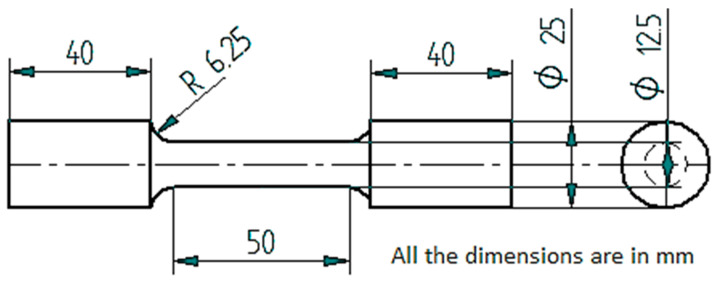
Tensile test specimen as per ASTM E8-95 standards.

The composites are characterised for tensile characteristics, which are prepared in accordance with the ASTM E8-95 standards (Figure 4), with a gauge diameter of 12.5 mm and a gauge length of 62.5 mm.

The tensile test specimens were characterised using a FINE INSTRUMENTS (Miraj, Maharashtra State, India) make TFUN-600 Universal Testing Machine with a strain rate of 0.00025/s (1.5%/min) available at MVJ College of Engineering, Bengaluru, Karnataka State, India, and the values of the tensile test were tabulated.

### 2.3. SEM and Elemental Analysis

The microstructural evaluation of the specimens was accomplished using a Hitachi make SU-8000 series SEM (Tokyo, Japan) available at the University Visvesvaraya College of Engineering, Bangalore University, Bengaluru, Karnataka State, India, with a scanning electron voltage of 15 keV at magnifications of 500× to study the particle distribution of the reinforcements in the matrix; in addition, the elemental analysis from the energy dispersive spectrum was performed using the silicon drift detector with the detection area of 100 mm^2^ to substantiate the findings by evaluating the presence of reinforcements. The SEM image depicts particle distribution in the matrix phase, while the EDS gives the elemental point analysis to significantly understand the homogenous dispersion of the SiC and fly ash flakes in the matrix facilitated by stir casting and thermal treatment.

### 2.4. Taguchi Method

Taguchi’s method is a useful statistical tool for studying the optimisation of the process. The study encompassed a design of experiments based on the orthogonal arrays (OA) and subsequent simplification of the experimental plan and the feasibility study involving the interaction between different parameters selected for the experimental trials. In the present work, optimisation studies were carried out in Minitab software based on the “larger is better” condition for ultimate tensile strength (UTS) and yield strength (YS), and “smaller is better” condition for the % elongation; the characteristic formulae for both conditions for each factor level combination are given in Equations (1) and (2), respectively.

(1) $$For\;“Larger\;is\;Better\;Condition”,\;S/N=-10*log\left(\Sigma \left(1/Y^{2}\right)/n\right)$$

(2) $$For\;“Smaller\;is\;Better\;Condition”,\;S/N=-10*log\left(\Sigma \left(Y^{2}\right)/n\right)$$

where *Y* = responses for the given factor level combination and *n* = number of responses in the factor level combination.

### 2.5. ANN Modelling

A neural network is a layered computer program with layers linked to nodes, and it resembles the network architecture of neurons of the human brain. Artificial neural network (ANN) can be taught to identify patterns, categorise information, and forecast future outcomes based on data [34]. The data are divided into abstraction layers by a neural network. The action of an artificial neural network is determined by how its individual elements are related and the weights of those links [35]. These weights are changed automatically, while training based on a learning rule until the neural network completes the task accurately. In the present work, one of the objectives formulated was to develop an ANN model that can predict the UTS in the experiment.

## 3. Results and Discussions

The characterisation of the composites was accomplished to evaluate the tensile properties and determine the variation of these properties with temperature and wt.% of reinforcements.

### 3.1. SEM and Elemental Analysis

**Figure 5:**
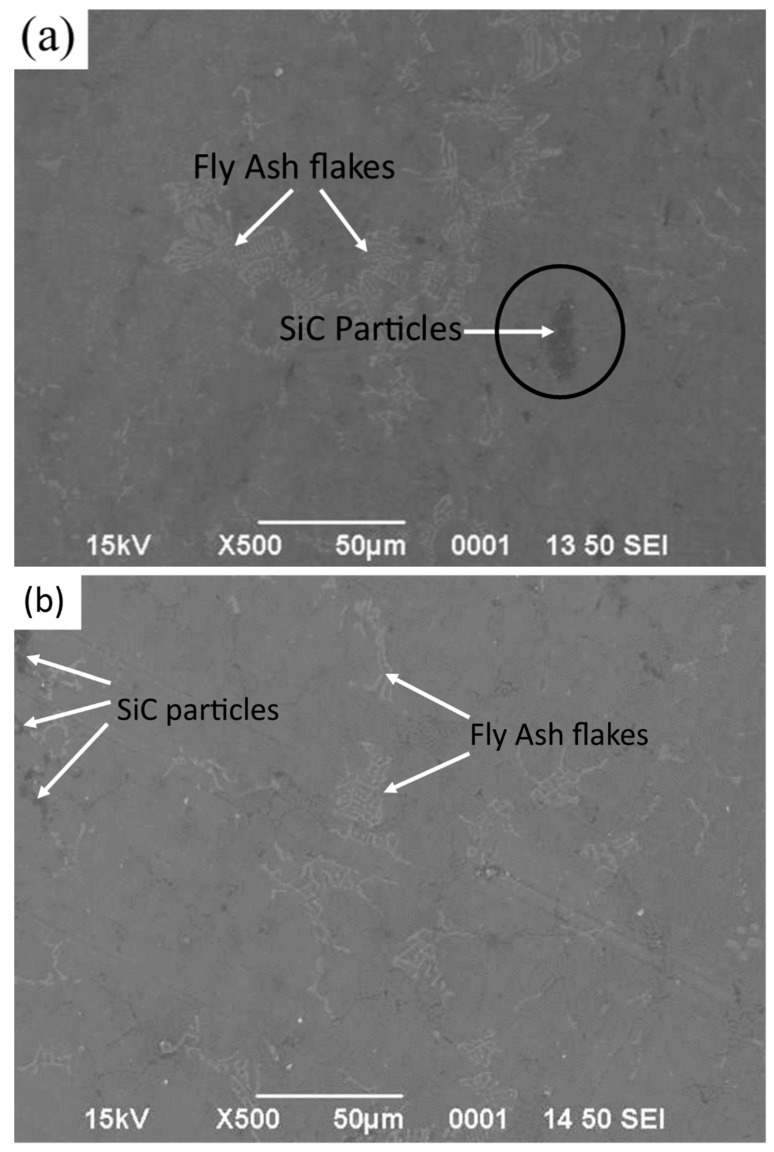

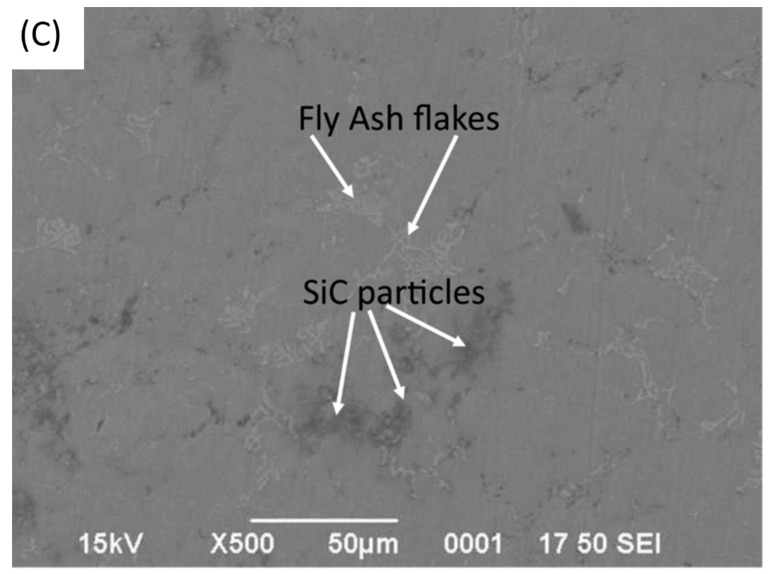
SEM image depicting the distribution in (**a**) AA 5083/5 wt.% SiC/2 wt.% fly ash, (**b**) AA 5083/5 wt.% SiC/4 wt.% fly ash, and (**c**) AA 5083/5 wt.% SiC/6 wt.% fly ash composites.

The SEM images were analysed to examine the microstructure and the elemental distribution using the energy dispersive spectrum of the chosen region of interest. The SEM images in Figure 5a–c depict the microstructure of AA 5083 alloy reinforced with 2, 4, and 6 wt.% fly ash and 5 wt.% SiC subjected to post-treatment at a temperature of 160 °C. Figure 5a depicts the micrograph of the composite with the agglomeration of SiC at some localised regions, while Figure 5b demonstrates the uniform distribution of the SiC due to the inoculation brought about by fly ash flakes. However, Figure 5c depicts a completely homogenous distribution of the reinforcements attributed to accelerated inoculation brought about by the increased addition of fly ash flakes and the post-exposure treatment at 160 °C. The SEM images thus depict the influence of inoculation on the homogenisation brought about by the processing and subsequent post-treatment. In this way, the uniform distribution of the particles brought about by the inoculation and heat treatment improve the tensile properties of the composites.

**Figure 6:**
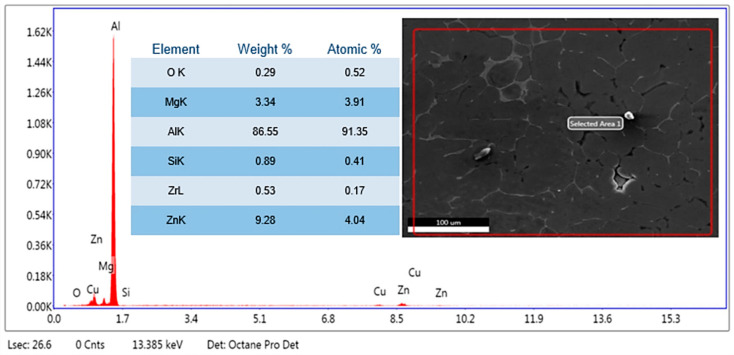
EDS of AA 5083/5 wt.% SiC/2 wt.% fly ash.

**Figure 7:**
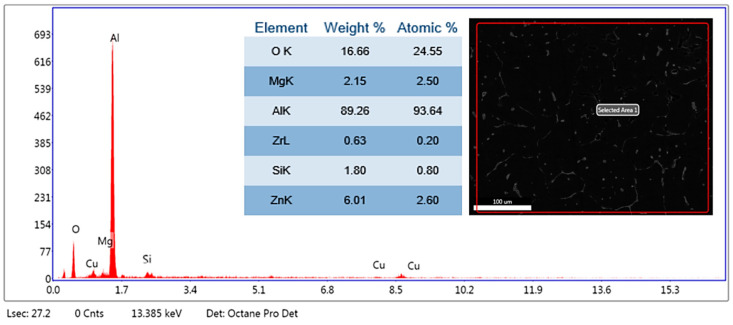
EDS of AA 5083/5 wt.% SiC/4 wt.% fly ash.

**Figure 8:**
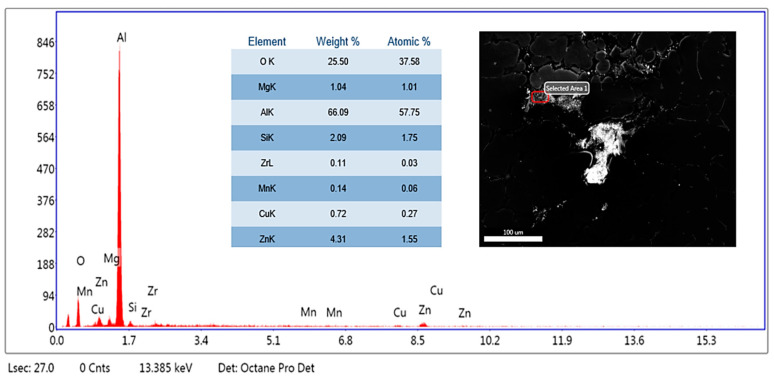
EDS of AA 5083/5 wt.% SiC/6 wt.% fly ash.

Further, the EDS of the composites clearly affirm the presence of Si, O, and Al-Mg elements in the composite, which ascertain the presence of matrix of aluminium AA 5083, and the SiC and fly ash reinforcements. Figure 6 shows the EDS spectrum of aluminium AA 5083 with 5 wt.% of SiC particulates and 2 wt.% of fly ash flakes, while Figure 7 depicts the EDS spectrum of aluminium AA 5083 with 5 wt.% of SiC particulates and 4 wt.% of fly ash flakes, which validates the inoculation. Essentially, the presence of oxygen indicates the aluminates and silicates of the fly ash reinforcements. Figure 8 depicts the EDS spectrum of aluminium AA 5083 with 5 wt.% of SiC particulates and 6 wt.% of fly ash flakes. The presence of SiC particulates as Si and C components and the presence of elemental oxygen confirm the uniform distribution of the reinforcements in the AA 5083 matrix brought about by the role of fly ash as an inoculant.

The EDS spectrums (Figure 6, Figure 7 and Figure 8) clearly depict the distinct peaks corresponding to the Al and Si, followed by the peaks corresponding to the elemental magnesium alongside the traces of other alloys ascertaining the fact that the aluminium alloy used for the matrix phase is basically of AA 5XXX series, which possess magnesium as the major alloying element and elemental oxygen components from the alumina (Al_2_O_3_) and silicates (SiO_2_) in the fly ash particulates.

### 3.2. Results of Tensile tests

➢ Ultimate Tensile Strength (UTS)

**Figure 9:**
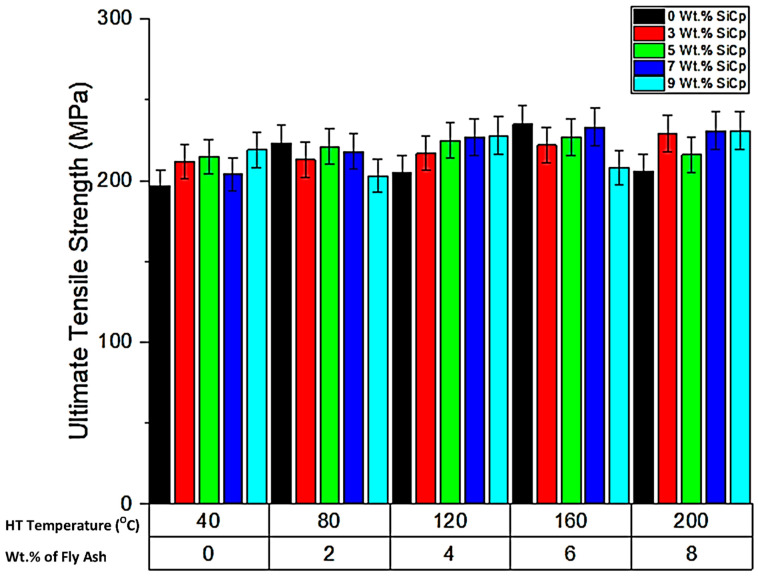
Variation of UTS for different experimental conditions.

**Figure 10:**
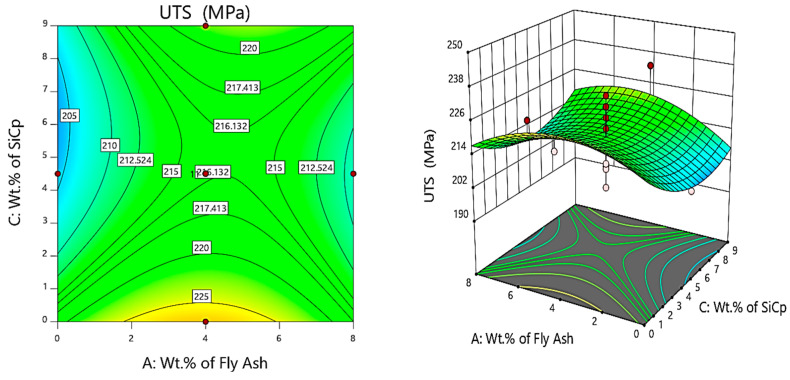
Contour and 3D surface plots for variation of UTS with varying wt.% of SiC and fly ash.

**Figure 11:**
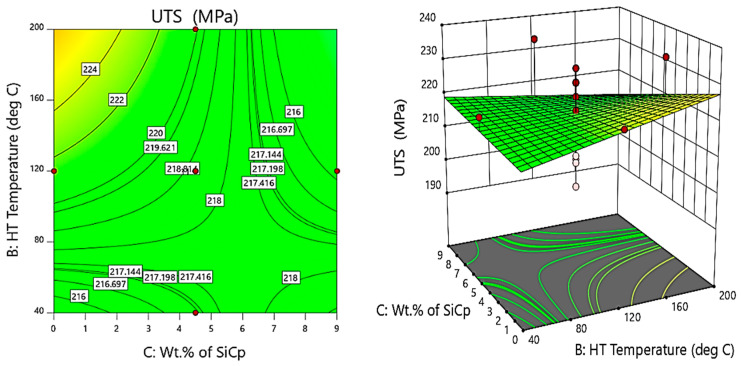
Contour and 3D surface plots for variation of UTS with varying wt.% of SiC and HT temperature.

**Figure 12:**
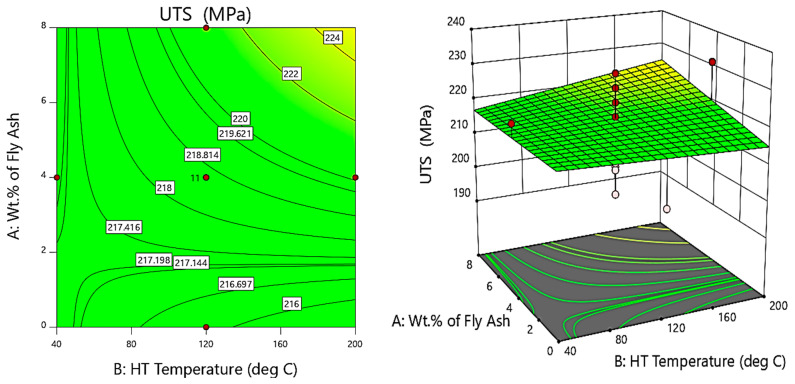
Contour and 3D surface plots for variation of UTS with varying wt.% of fly ash and HT temperature.

The comparative evaluation of the ultimate tensile strength of the composite specimens for different compositions of the reinforcements and heat treatment temperatures is given in Figure 9, Figure 10, Figure 11 and Figure 12. From the graph (Figure 9), it is evident that the ultimate tensile strength of the composites increases with the increase in the wt.% of fly ash due to inoculation accelerated by the thermal exposure of the composites in the post-treatment condition up to a temperature of 160 °C, as ascertained by the findings of the Kumar et al. [36], who reported the findings related to the influence of fly ash reinforcements on the tensile strength of Al/3Cu/8.5Si and drew similar inferences of increase in the strength with the increase in wt.% of fly ash attributed to close packing of the reinforcements in the matrix. However, the increase in the thermal exposure temperature to 200 °C leads to a slight decrease in the ultimate tensile strength which can be attributed to the agglomeration of the reinforcements at certain regions in the matrix at an elevated temperature of exposure, as reported in the findings of Kok et al. [37] about the effect of thermal exposure on the Al_2_O_3_ particle dispersions in the matrix; the Al_2_O_3_ particles dispersed in the matrix phase and exposed to a temperature of 150 °C had better strength characteristics, as compared to those aluminium composites with Al_2_O_3_ particle dispersions in the matrix subjected to a temperature above 150 °C which is in line with the findings of the present work.

Further, the contour and 3D surface plots (Figure 9) depict that the inoculation brought about by the fly ash reinforcements will enhance the strength of the composites, due to the strong bonding and inherent coring of the reinforcements with the matrix brought about by the thermal exposure. The inferences are based on those of Gireesh et al. [38], who reported an increase of the tensile strength of the Al 6061 reinforced with SiC and Al_2_O_3_ by 8.5%, while an increase in yield strength by 36.8% mainly due to the adhesive bonding between the matrix and reinforcements attributed to uniform mixing and distribution of the reinforcements.

Santhosh et al. [39] reported the findings of tensile characterisation of aluminium AA 5083/SiC/fly ash composites and compared the results with the base alloy. They distinctly identified the need to fabricate high-performance hybrid aluminium composites and reported that the mechanical properties particularly the tensile strength improves up to a certain extent, i.e., 3 to 5 wt.% of SiC; however, beyond this range, the tensile strength showed a decreasing trend. This is addressed by the present research, wherein the tensile strengths show continuous improvement because of the controlled dispersion of the SiC reinforcements alongside fly ash in the matrix and the T6 thermal treatment, due to which the solute elements precipitate in the Al solid solution, facilitating strong bonding the matrix and the reinforcements. The trend observed with respect to the increase in the tensile characteristics from the present work substantiates the need for thermal treatment for the improvement in the characteristics of the composites owing to better bonding and interstitial strengthening.

The influence of heat treatment on the strength of these composites is of significant importance to enhance the characteristics of the composites for real-time engineering applications. In this regard, the contour plots and surface plots help us to understand the influence of heat treatment on the ultimate tensile strength of the composite materials. The contour and 3D surface plots in Figure 11 give the variation of the ultimate tensile strength (MPa) with heat treatment and wt.% of SiC. From the 3 D surface and contour plots, it is evident that the ultimate tensile strength is maximum for composite specimens with 9 wt.% of SiC, exposed to a treatment temperature of 160 °C, i.e., the specimens after successful heat treatment, especially after T6-grade treatment and thermal exposure for a duration of 24 h in oven conditions at 160 °C, will form strong bonds with enhanced toughness, owing to micro coring and granular segregations.

Further, Figure 12 shows the contour and 3D surface plots for the variation of the ultimate tensile strength (MPa) with heat treatment and wt.% of fly ash. From the plots, it is evident that the ultimate tensile strength is maximum for composite specimens with 6 wt.% of fly ash, exposed to a treatment temperature of 160 °C, i.e., the composite specimens after successful heat treatment, especially after T6-grade treatment and thermal exposure at a temperature of 160 °C, will form strong bonds between the Al atoms and the silicates present in the fly ash. It is, therefore, evident from the graphs that the ultimate tensile strength is dependent on the wt.% of fly ash and the heat treatment temperature [39].

The findings are in line with the research outcomes of Tiwari et al. [40], who studied the influence of heat treatment on mechanical characteristics of aluminium–fly ash composites. Their findings indicate that the varying wt.% of fly ash and heat treatment of the composite specimens enhance the mechanical properties attributed to them through modification of the crystalline structure of the composites, followed by the varying wt.% and size of the fly ash particles. The investigations carried out by Chen et al. [41] on the influence of heat treatment on the microstructure and mechanical properties of TiB_2_ reinforced Al 2024 composites provided a fundamental basis for the thermal exposure of the composites, and the results reveal that the addition of TiB_2_ results in significant grain refinement with the random orientation of θ-Al_2_Cu phases.

➢ Yield Strength

**Figure 13:**
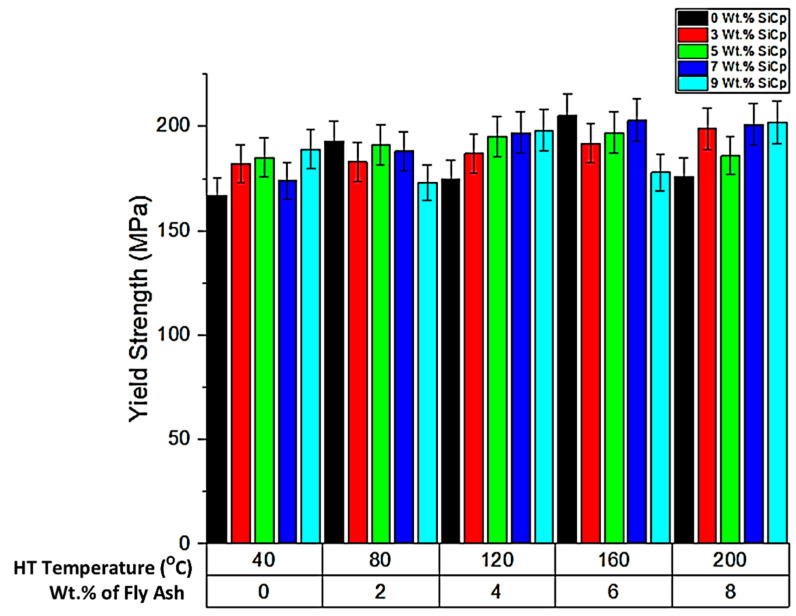
Yield strength for different experimental conditions.

**Figure 14:**
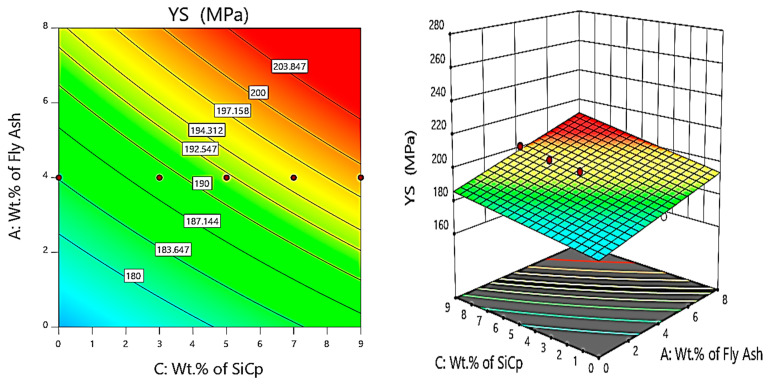
Contour and 3D surface plots for variation of YS with varying wt.% of SiC and fly ash.

**Figure 15:**
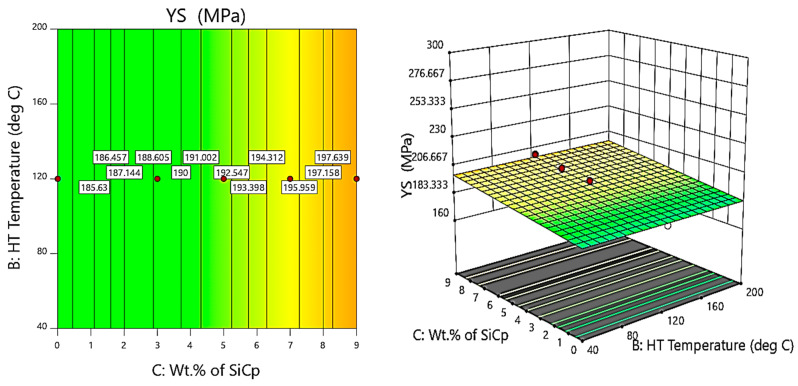
Contour and 3D Surface plots for variation of YS with varying wt.% of SiC and HT temperature.

**Figure 16:**
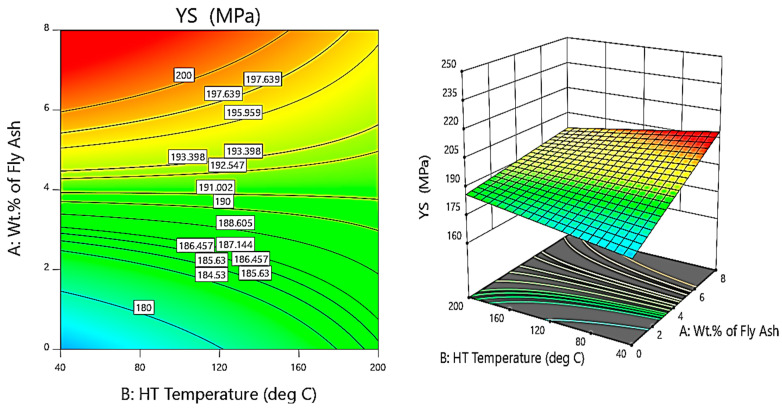
Contour and 3D Surface plots for variation of YS with varying wt.% of fly ash and HT temperature.

The comparative evaluation of the yield strength of the composite specimens for different heat treatment temperatures and composition of reinforcements is presented in Figure 13, Figure 14, Figure 15 and Figure 16. From the graph, it is evident that the yield strength of the composites increases with the inoculation, resulting in grain packing and bonding between the atoms and thereby increasing the yield strength which is further facilitated by the thermal exposure of 160 °C, attributed to the increase in the stiffness of the material; however, beyond 160 °C, there will be a slight reduction in the yield strength. The findings are supported by the research of Santhosh et al. [39], wherein the inclusion of fly ash brought about the inoculation in the matrix, thereby leading to coherent bonding and interstitial micro coring. The process of inoculation is further accelerated in the present work due to the homogenisation of the fly ash particles in the matrix phase brought about by uniform stirring and post-treatment thermal exposure. Subrahmanyam et al. [42] carried out the inoculation of aluminium alloy using agricultural waste rice husk ash. The researchers reported that the mechanical characteristics of MMCs performed exceptionally better with the inclusion of RHA in aluminium alloy. The microstructure of the composites fabricated by them with 8% RHA particles exhibited exceptional mechanical characteristics and uniformly distributed particulates.

Further, the contour and 3D surface plots (Figure 14, Figure 15 and Figure 16) are considered for the variation of yield strength (YS) with the weight percentage of SiC and fly ash and the heat treatment (HT) temperature. It is clearly evident from the contour and surface plots that the composites synthesised with 6 wt.% of fly ash, 9 wt.% of SiC, and exposed to a temperature of 160 °C have a major influence on maximising the yield strength of the AA 5083/SiC/fly ash composites.

From the contour and 3D surface plots for variation of YS with varying wt.% of reinforcements at different heat treatment temperatures, it is clearly evident that the reinforcements and heat treatment increases the yield strength of the composites, attributed to Orowan strengthening facilitated by precipitation hardening. Shin et al. [43] identified the microstructural evolution and strengthening mechanisms in Al/SiC composites en route the liquid pressing process, subsequently followed by the heat treatment process, which helps in increasing the strength due to solution heat treatment and artificial ageing, especially due to the formation of Mg_2_Si precipitates attributed to precipitation hardening.

➢ % Elongation

**Figure 17:**
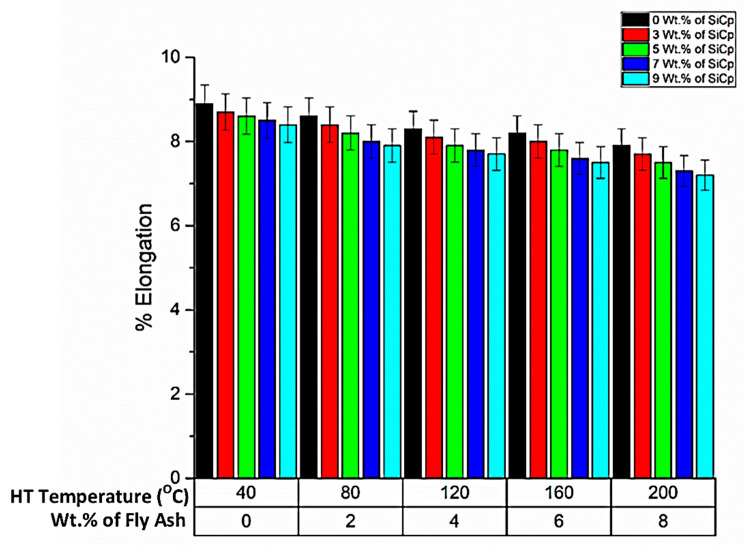
%Elongation for different experimental conditions.

% Elongation is an important attribute for determining the ductility of the composites; specifically, it gives an overview of the length up to which the material elongates in the plastic zone before fracturing. The bar chart in Figure 17 lists the % elongation that the composites undergo before failure. The % elongation of the base alloy (AA 5083) specimens are relatively higher than the composites, attributed mainly due to the lack of stiffness and the lack of the ability to absorb the applied loads in the plastic zone before failure; however, the percentage elongation decreases with the increase in the reinforcements, especially the SiC reinforcements, which are hard ceramic particles that increase the hardness and strength, causing embrittlement in the composite phase. Similarly, fly ash particles and heat treatment also lead to strong bonding between the Al-C compounds, with the reaction between the atoms accelerated by the inoculation of the Si atoms from the fly ash in the Al matrix. Further, the formation of Al-Si-C bonds along with the Mg atoms along the periphery of the compounds leads to the strengthening of the composites and an increase in the stiffness of the composites, with increased embrittlement between the atoms.

The comparative evaluation of the % elongation of the composite specimens for different heat treatment temperatures is interpreted from the graph (Figure 17). From the graph, it is evident that the % elongation of the composites decreases with the increase in the wt.% of SiC and fly ash and the heat treatment temperature, mainly attributed to the coherent bonding and interstitial micro coring and increase in the stiffness of the material. This is also supported by the inferences of Florián-Algarín et al. [44], who concluded that, as the strength increases, the elongation decreases; this is due to the fact that the increase in strength, especially the increase in ultimate tensile strength, yields in increased strength, and Young’s modulus significantly increases the stiffness of the material, thereby decreasing the tenacity related to the % elongation (ductility). However, at the thermal exposure temperature of 160 °C, there is a slight increase in the % elongation, due to the critical softening of the material occurring consequent to the thermal deformation, beyond which the % elongation decreases due to embrittlement accelerated by the strain hardening, as noted in the works of Lu et al. [45] on the influence of hard ceramic reinforcements in enhancing the mechanical strength of the composite materials. They studied the impact of reinforcements in enhancing the damping characteristics of the composite materials, especially attributed to the synthesis of composites in controlled environments.

**Figure 18:**
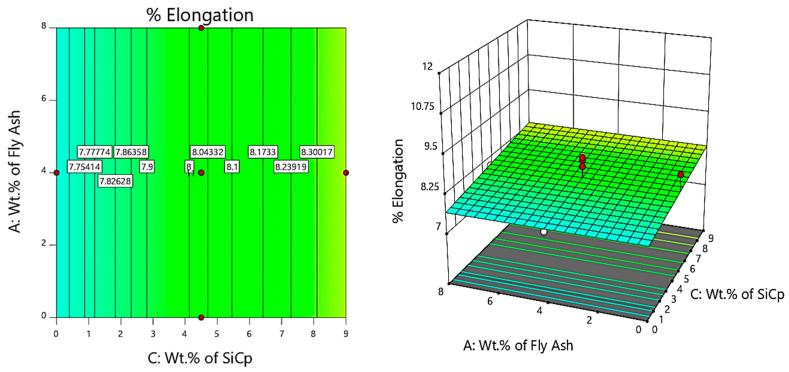
Contour plot for variation of % elongation with varying wt.% of fly ash and SiC.

**Figure 19:**
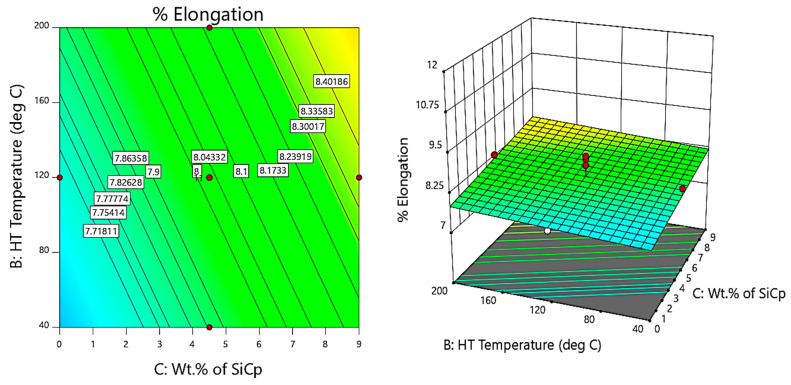
Contour plot for variation of % elongation with varying wt.% of SiC and HT temperature.

**Figure 20:**
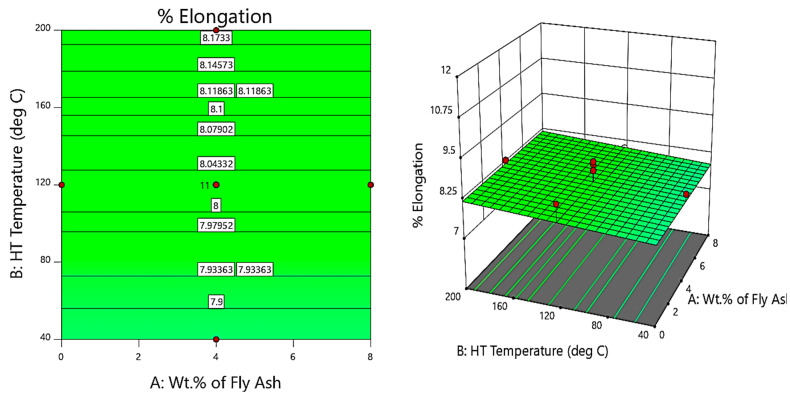
Contour and 3D Surface plots for variation of % elongation with varying wt.% of fly ash and HT temperature.

Further, the contour and 3D surface plots (Figure 18, Figure 19 and Figure 20) depict that the inclusion of the reinforcements reduces the ductility of the composites due to the strong bonding facilitated by the inclusion of reinforcements that leads to the process of precipitation hardening, accelerated by the heat treatment, i.e., as the temperature of thermal exposure increases, the hardness and strength of the composites increase due to micro coring and interstitial bonding, while the ductility decreases due to embrittlement and crack initiation and rapid propagation along the 45° plane. Although the ductility decreases with heat treatment up to 160 °C, the % elongation at 160 °C slightly increases with the slightest improvement in ductility, due to thermomechanical softening and easy deformation [46].

From the contour and 3D surface plots for variation of % elongation with varying wt.% of reinforcements at different heat treatment temperatures, it is clearly evident that the reinforcements and heat treatment decreases the % elongation of the composites, thereby reducing the ductility of the composites attributed to strain hardening and embrittlement. The findings are in line with the results reported by Gan et al. [47]. The researchers studied the processing of composite materials and analysed the effect of particulates on the ductility of the composites developed; they indicated the decrease in ductility with the agglomeration of the hard ceramic particulates and suggested the friction processing of composites as one of the methodologies of fabrication of composites for better mechanical properties.

➢ Optimisation Studies for Ultimate Tensile Strength (UTS)

**Figure 21:**
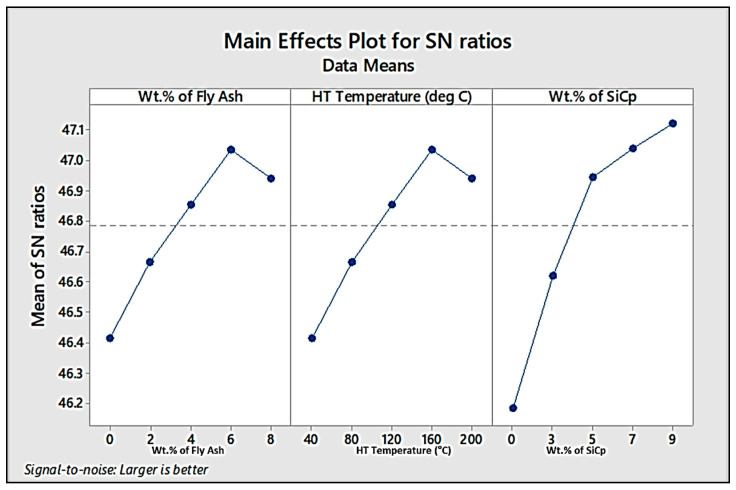
Main-effect plot for S/N ratios for UTS.

**Figure 22:**
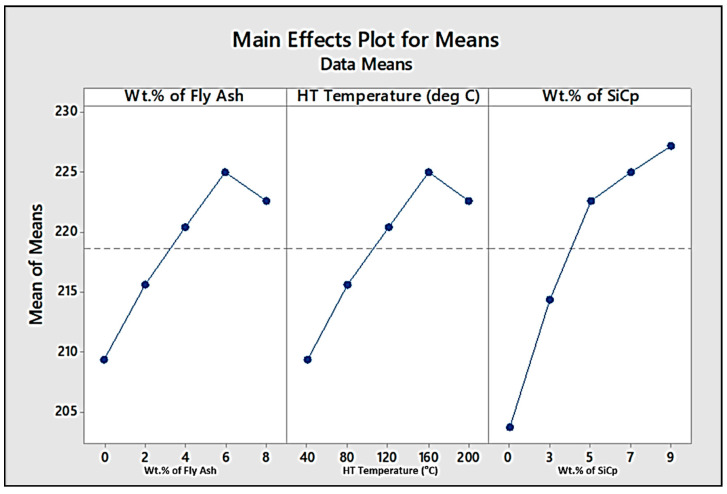
Main-effect plot for means for UTS.

Optimisation studies by Taguchi methods were carried out to find the optimised wt.% of fly ash and SiC and the temperature of the exposure for maximising the UTS of the composite specimens fabricated en route stir casting. Additionally, the optimisation results give us an overview of the influence of the reinforcements and heat treatment on the ultimate tensile strength of the composites developed. Table 6 and Table 7 provide the response tables for S/N ratios and means, while Figure 21 and Figure 22 show the main-effect plots for S/N ratios and means for the UTS.

From the response tables and main-effect plots for S/N Ratios and means, it is evident that the wt.% of SiC has a major influence on the UTS, followed by the wt.% of fly ash and heat treatment temperature. Among these factors, level 4 (6 wt.%) for fly ash and level 4 (160 °C) for HT temperature are found to be the optimised set of parameters for maximising the UTS, while the inclusion of silicon carbide in the increments of 2 wt.% is found to outrightly improve the UTS, beginning from 3 wt.% up to the maximum limit of 9 wt.% chosen for the present work.

Parasuraman et al. [48] studied the optimisation of machinability of AA 7075/TiB_2_ composites; the parametric studies helped optimise the cutting forces and surface roughness during the drilling of composites, and the methodology adopted by the authors are in line with the statistical studies carried out in the present work to optimise the process parameters for stir casting the composites, subsequently followed by post-treatment to obtain maximum strength.

Park et al. [49] reported the interface and synthetic reaction between the atoms and subatomic packings in the matrix and their effect on the characteristics of the composite materials developed. It has been observed, from their studies, that the interface reaction is accelerated by the grain growth epitaxy due to the atomic reagents in the form of material inoculants, which leads to improved tensile strength in the composites.

## 4. Results of ANN Modelling

**Figure 23:**
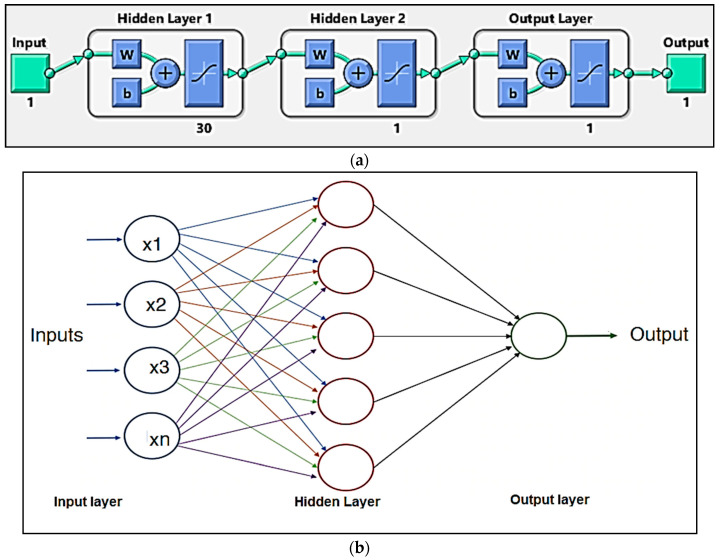
(**a**) Hidden layers and (**b**) ANN architecture.

The ANN method is considered to be the optimal option for any problem involving nonlinear and complex relationships between the variables. The neurons are organised via layers in the multi-layer perceptron (MLP) structure, with one input neurons layer, another output neuron layer, and one or more hidden layers. The hidden layers are made up of numerous interconnected neurons, as shown in Figure 23a. The ANN architecture comprising of the input, output, and hidden layers is shown in Figure 23b.

**Figure 24:**
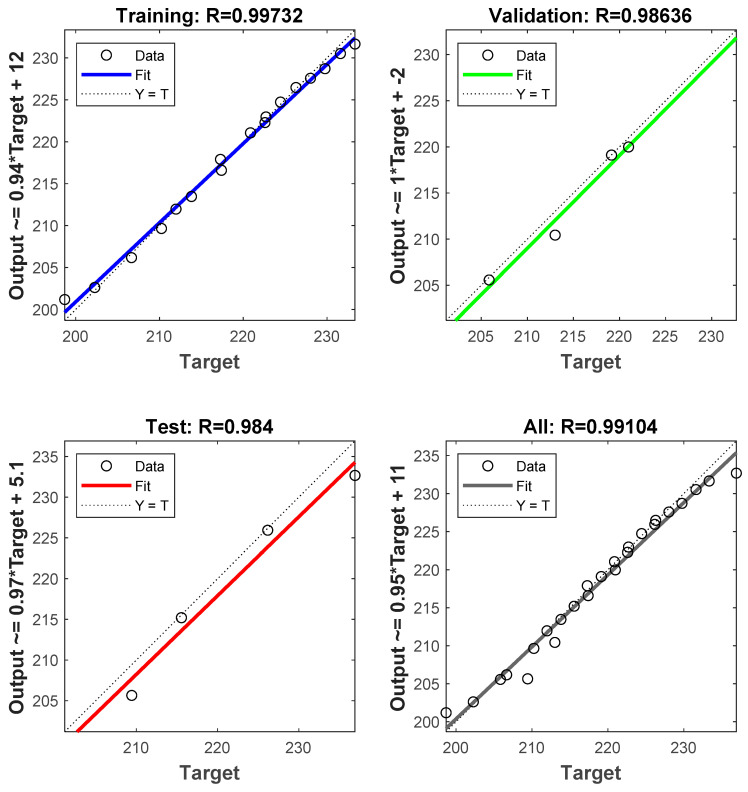
Optimal performance was found at 30 neurons in the hidden layer.

**Figure 25:**
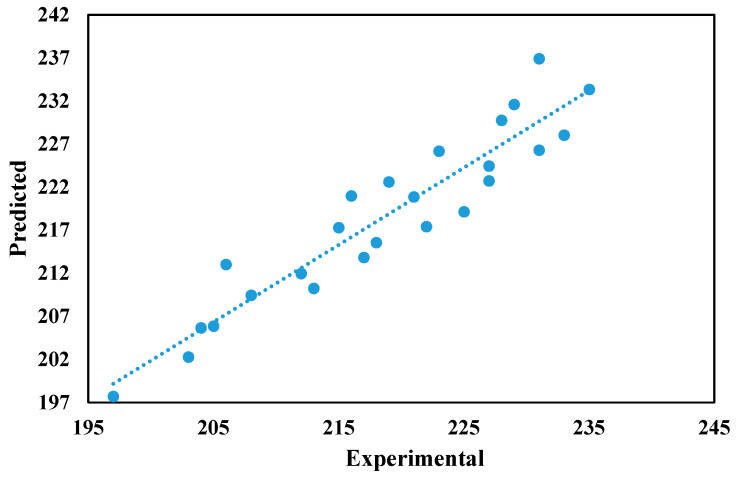
ANN modelling results vs. experimental results (UTS).

The ANN used 520 experimental results, split into 3 segments: the training set (65% data), the test set (25% data), and the validation set (10% data). The importance of determining the best ANN architecture is critical because it has a significant impact on the results [50,51,52]. The optimisation of ANN variables is achieved by minimising the mean square error (MSE) after examining a large number of distinctly configured neural networks [53]. The appropriate ANN framework that was chosen in this analysis has 30 neurons in the hidden layer for the prediction of UTS as shown in Figure 24. A tangent sigmoid transfer feature operates in the hidden layer [54,55,56]. A linear transfer function and the ‘Levenberg–Marquardt’ learning algorithm are used in the output layer [57,58]. In Table 8, the correlation coefficient of ANN models is shown for testing, training, and validation data. The test data were compared to the expected UTS data obtained from ANN (Figure 25, Table 9). As can be seen, ANN’s estimation of the results is significantly better.

The ANN model thus provided a framework for prediction of the tensile strength characteristics of the composites developed for different compositions of the reinforcements and the heat treatment conditions, thus evolving a scientific database for further experimental trials on the composites for need-based fabricate on of components from the given combination of constituents for automotive and aerospace applications [59].

## 5. Conclusions

The review of the outcomes of the experiments and the statistical validations yielded optimum parameters for maximising the strength of the composites developed. The distribution of the reinforcements was ascertained from the SEM images. The composites subjected to T6 tempering with solution treatment, subsequently followed by the precipitation hardening, enhanced the characteristics, especially the tensile and yield strength of the composites, facilitated by the thermal exposure. However, the percentage elongation of the composites was reduced significantly with the increase in reinforcements owing to inherent bonding, which was further accelerated by the T6 treatment.

The findings for tensile strength were further optimised using Taguchi methods, and the optimised set of parameters for obtaining maximum ultimate tensile strength, yield strength, and stiffness for the composites were determined. The ANN modelling yielded modelling coefficients achieved by minimising the MSE after examining a large number of distinctly configured neural networks; the appropriate ANN framework that was chosen in this analysis has 30 neurons in the hidden layer for the prediction of UTS. The test data were compared to the expected UTS data obtained from ANN, and it is evident from the findings that the ANN’s estimation of the results is accurate, with the error % between the test results and the predictions less than +/−2%.

Thus, the findings of the experiment and statistical validations provide optimised set of parameters for fabrication and post0treatment of AA 5083/SiC/fly ash composites for high-performance applications, especially as structural components in automotive and aerospace domains, wherein the composites have to possess high tensile, yield strength, and stiffness. The present work can be extended to the fabrication of real-time applications based on the post-treatment conditions optimised for the set of parameters considered in this research.

## Figures and Tables

**Table 1:** Composition specification of the AA 5083 alloy in wt.% of elements.

Elements in the Alloy	% Present in the Alloy
Cu	0.15
Zn	0.20
Ti	0.20
Cr	0.1
Mg	4.5
Si	0.45
Fe	0.45
Mn	0.7
Al	Balance

**Table 2:** Properties of the matrix material (AA 5083).

Property	Value
Melting point Density	272 °C 2655 kg/m^3^
Modulus of elasticity	71.95 GPa
Thermal conductivity	120.6 W/mK
Electrical resistivity	5.6 × 10^−8^ Ωm
Thermal expansion	25 × 10^−6^/K

**Table 3:** Composition table of fly ash (C type).

Composition	Requirements (ASTM C618), %
SiO_2_, Al_2_O_3_, Fe_2_O_3_ (min)	50–55
SO_3_, max	5–7.5
Moisture content, max	4–5
Loss on ignition, max	5–7.5

**Table 4:** Property table of silicon carbide (SiC).

Formula	SiC
IUPAC ID	Silicon Carbide
Melting point	2730 °C
Density	3.24 g/cm^3^
Molar mass	40.11 g/mol

**Table 5:** Experimental conditions for different trials.

Trial Designation	wt.% of Fly Ash	HT Temperature (°C)	wt.% of SiC
L1	0	40	0
L2	0	40	3
L3	0	40	5
L4	0	40	7
L5	0	40	9
L6	2	80	0
L7	2	80	3
L8	2	80	5
L9	2	80	7
L10	2	80	9
L11	4	120	0
L12	4	120	3
L13	4	120	5
L14	4	120	7
L15	4	120	9
L16	6	160	0
L17	6	160	3
L18	6	160	5
L19	6	160	7
L20	6	160	9
L21	8	200	0
L22	8	200	3
L23	8	200	5
L24	8	200	7
L25	8	200	9

**Table 6:** Response table for signal-to-noise (S/N) ratios for ultimate tensile strength.

Larger Is Better			
Level	wt.% of Fly Ash	HT Temperature	wt.% of SiC
**1**	46.41	46.41	46.18
**2**	46.67	46.67	46.62
**3**	46.86	46.86	46.95
**4**	47.04	47.04	47.04
**5**	46.94	46.94	47.13
**Delta**	0.62	0.62	0.97
**Rank**	2.5	2.5	1

**Table 7:** Response table for means for ultimate tensile strength.

Larger Is Better			
Level	wt.% of Fly Ash	HT Temperature	wt.% of SiC
**1**	209.4	209.4	203.8
**2**	215.6	215.6	214.4
**3**	220.4	220.4	222.6
**4**	225.0	225.0	225.0
**5**	222.6	222.6	227.2
**Delta**	15.6	15.6	23.4
**Rank**	2.5	2.5	1

**Table 8:** Modelling coefficients for predicting the UTS.

Number of Hidden Layer	Neurons in Hidden Layer	Modelling Coefficient *R*^2^			
		Training Data	Validating Data	Testing Data	All Data
1	10	0.99712	0.98606	0.9964	0.9988
1	15	0.99720	0.98616	0.9968	0.9990
1	20	0.99724	0.98620	0.9972	0.9994
1	25	0.99728	0.98628	0.9978	0.9998
1	**30**	**0.99732**	**0.98636**	**0.9984**	**0.99104**
1	40	0.99730	0.98631	0.9980	0.99100

**Table 9:** ANN predictions for UTS.

Trial Designation	Experimental	Predicted	% Error
L1	197	197.6924	0.35
L2	204	205.6597	0.81
L3	212	211.9711	−0.01
L4	215	217.2826	1.06
L5	219	222.5941	1.64
L6	203	202.2725	−0.36
L7	213	210.2397	−1.30
L8	218	215.5512	−1.12
L9	221	220.8626	−0.06
L10	223	226.1741	1.42
L11	205	205.8525	0.42
L12	217	213.8197	−1.47
L13	225	219.1312	−2.61
L14	227	224.4427	−1.13
L15	228	229.7542	0.77
L16	208	209.4326	0.69
L17	222	217.3998	−2.07
L18	227	222.7113	−1.89
L19	233	228.0227	−2.14
L20	235	233.3342	−0.71
L21	206	213.0126	3.40
L22	216	220.9798	2.31
L23	231	226.2913	−2.04
L24	229	231.6028	1.14
L25	231	236.9142	2.56

## Data Availability

The data presented in this study are available on request from the corresponding authors.
